# Evaluation of Adhesion and Morphology of Human Osteoblasts to White MTA and Portland Cement

**Published:** 2007-10-02

**Authors:** Maryam Bidar, Jalil Tavakkol Afshari, Fatemeh Shahrami

**Affiliations:** 1 *Department of Endodontics, Dental Research Center, Dental School, Mashad University of Medical Sciences, Mashad, Iran*; 2 *Department of Immunology, Medical School, Mashad University of Medical Sciences, Mashad, Iran*

**Keywords:** Human Osteoblasts, MTA, Portland Cement, Root End Filling Material

## Abstract

**INTRODUCTION:** Osteoblasts and periodontal ligament cells are major cells for wound healing after root end resection. The interaction of osteoblasts with filling materials could play a critical role in healing of surgical lesion. Adhesion and spreading of cells on material surface are the initial phase for cellular function. The purpose of the present study was the evaluation of morphology and attachment of human osteoblasts in present of white MTA, Portland cement (PC) and IRM as root end filling and perforation repair materials.

**MATERIALS AND METHODS:** The human osteoblasts (MG-63 cell line) were prepared from Iranian Pasteur Institute; Cellular Bank, were grown in RPMI 1640 medium. The testing materials were mixed according to the manufacture's instruction, inserted in to the wells of 24-well flat-bottomed plate, and condensed to disk of 1mm thickness and 1×1mm diameter. Cells were added to the materials after two weeks. During 1,3,7 days intervals, the disk of materials along with cells were grown on their surface, examined by a scanning electron microscope (SEM). We used of IRM as negative group.

**RESULTS:** Results showed that after 7 days many of osteoblasts were attached on the surface of white MTA and PC and appeared partially round or flat. The cells appeared round with no attachment and spreading in conjunction with IRM.

**CONCLUSION:** The results indicate that human osteoblasts have a favorable response to white MTA and Portland cement compared with IRM.

## INTRODUCTION

In some cases after root canal therapy and during repair of perforations and after retreatment, if there is a persistence lesion, we need to do surgical treatment and access to lesion through the root end. The process include exposure of root end and resection of it, preparation of cavity and place one material in cavity in contact with vital tissue of periapical (1).

One of the final aims in root canal therapy is regeneration of attachment apparatus and because of root end filling materials are in direct contact with vital connective tissue and bone, true regeneration of these tissues depends on normal function of osteoblasts, fibroblasts and cementoblasts for bone formation, PDL and cementum. Therefore used materials must be biocompatible and nontoxic and able to stimulate healing (1).

The commonly used root-end filling materials are Amalgam, IRM (Intermediate Restorative Material), super EBA**,** composite resin, Glass ionomer and new material as Mineral Trioxide Aggregate (MTA) and Portland cement (1).

Histological reports have indicated that new cementum may be formed adjacent to a few dental materials when placed in contact with periodontal tissue (2). These materials include MTA, composite resin, and hydroxyapatite (2).

Recently MTA is used extensively as root-end filling material (1). Histological studies have shown noticeable bone regeneration with MTA (1). New research studies showed that if MTA placed in a synthetic tissue fluid such as phosphate buffer saline (PBS) hydroxyapatite could form over the MTA (3,4) and because the pH of MTA is similar to calcium hydroxide (CaOH2), this materials could be used in formation of hard tissue (5).

In 2002 the gray MTA was substituted by the new white MTA Proo Root MTA, which is a powder consists of fine hydrophilic particles that sets in the presence of moisture (6). The setting time is about 4h and 3h after mixing the PH value is 12.5 (6).

The basic raw materials of Portland cement are lime (CaO), silica (SiO2), alumina (AL2O3), and iron oxide (Fe2O3). In the manufacturing process, they are crushed, ground, proportioned for the desired composition and then heated up to 1400-1600c.Added gypsum (CaSO4.4H2O) controls the setting time of the cement. The resulting product consist of tricalcium silicate dicalcium silicate, tricalcium aluminate and tetracalcium alumino ferrite (7). The aim of our study was evaluating of human osteoblasts response to white MTA and Portland cement as root-end filling materials by SEM.

## MATERIALS AND METHODS

The selected cell line MG-63 cells derived from human osteosarcoma were purchased from National Cell Bank of Iran (NCBI) and grown in RPMI-1640 medium with 10% FCS and 1% antibiotic-antimycotic (100 U/mL of penicillin, l00 ug/mL of streptomycin, and 1% amphotericin B) under standard cell culture conditions (37°C, 95% humidity, 95% air and 5% CO2). The culture medium was changed every 3 to 4 days.

Root-end filling materials used in this study were white MTA (ProRoot, Dentsply, Tulsa Dental, OK, USA), Portland cement (Type IV, Mashad Siman, Mashad, Iran) and IRM (Caulk, Dentsply, Milford, DE, USA).

Materials were mixed according to the manufacturer's instruction, and placed in 24-well flat-bottomed plates, and condensed to disks of ~1mm thickness and ~1x1 mm diameter. Cell culture medium (2cc/well) was put into the wells with the material disks and incubated at 37°C and 95% humidity. The medium was changed every day for 2 weeks. This allowed the materials to set and cytotoxic material to be eluted. After 2 weeks human osteoblasts were seeded into the wells at 4xl0^4^ cells per well. For each material to be tested, four samples were prepared. Normal cells were positive group and IRM group was negative group. Cells were adjacent to materials for 1, 3, 7 days. In total 48 samples were prepared. After incubation, the disks of root-end filling materials along with cells grown on their surface were fixed and prepared for evaluating by scanning electron microscope. For fixation, disks were washed three times with PBS, fixed with 2/5% glutaraldehyde for 1 h, and then post-fixed for 30min in 2% osmiumtetraoxide in the same buffer. The samples were dehydrated in ascending grade of ethanol (25% for 15min, and four times in 100% for 20 min each), immersed in hexamethyl disilazane for 30 min, and were then air-dried.

**Preparation for examination by scanning electron microscope: **After fixation samples sputter-coated with 15nm gold palladium (Polaron 5200. sputter coater) and were examined at ×100 and ×1000 magnification (SEM: LEO1450 VP, Germany).

**Methods of Evaluation** **: **Cells were observed under (SEM) for morphological alteration and evaluating of adhesion. If cells appeared flat or partially round and attached to surface, that material was biocompatible and cells have normal function. If cells are appeared totally round and detached from surface, that material is toxic and not biocompatible. In this study IRM was used as negative control group according to previous research study (1, 2, and 8). Normal osteoblasts that were not adjacent to any materials were considered as positive control.

## RESULTS

After 24 h in culture, osteoblasts attached and spread well on the surface of white MTA and Portland cement and were flat or partially round and adjacent to IRM were totally round and lost their attachment.

In positive group the cells were normal with attaching to surface. After 3 days in adjacent to white MTA and PC cells were flat or partially round and cells were attached.

**Figure 1:**
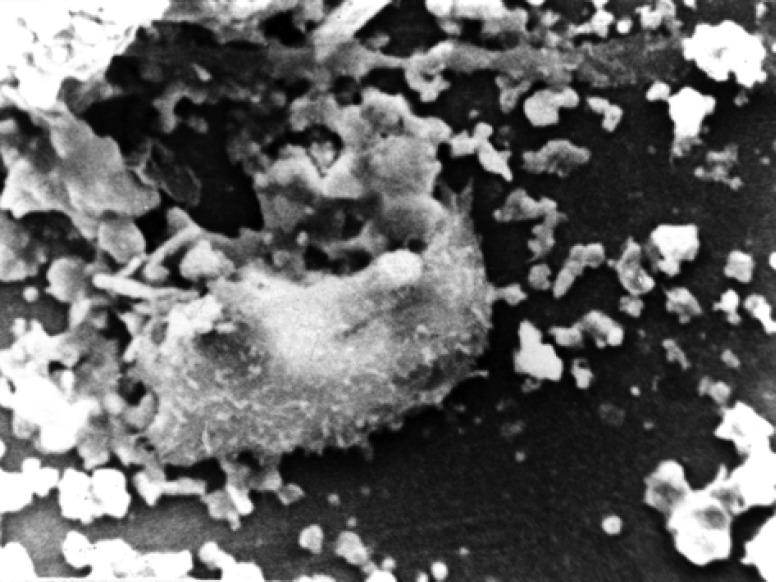
White MTA group in seventh day. Cell with attachment and normal morphology (×1000)

**Figure 2:**
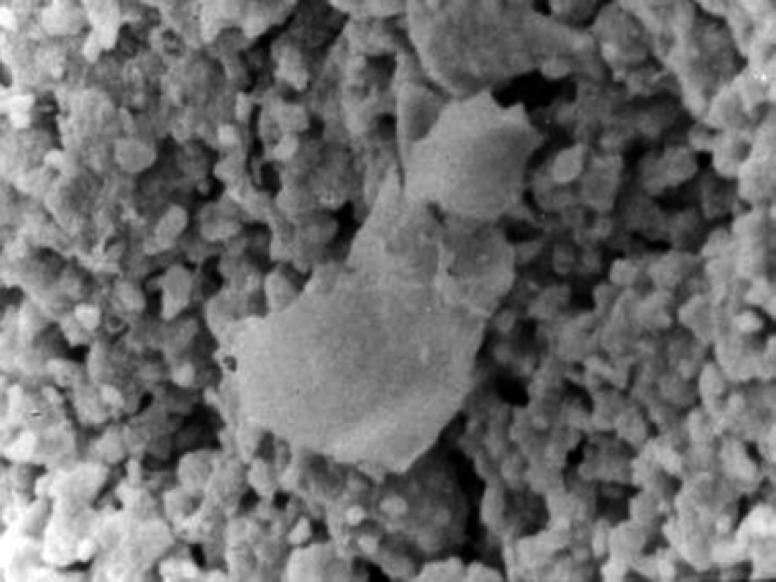
Portland cement group in seventh day. Cell attached to surface (×1000)

In seventh days osteoblasts on the surfaces of white MTA were flat with attachment (Figure 1) and surface on Portland cement was partially round with attachment (Figure 2). In negative group cells were totally round with no attachment.

## DISCUSSION

Retrograde root-filling materials should possess the highest biocompatibility when placed in direct contact with periradicular tissue. Safavi *et* *al.* described the quality and quantity of cell attachment to the retrofilling materials as an indication of the materials biocompatibility (9).

Zhu *et* *al.* suggested that cell adhesion and spreading on root-end filling materials could be used as criterion for evaluation of root-end filling materials (1). A disadvantage of commonly use in vitro biocompatibility testing system is that in such assay, only the cytotoxicicity is evaluated. Other factors such as the material’s physical structure and surface characteristics, known to influence the tissue response to the materials, are rarely considered. Studies evaluating the cytotoxicity of MTA have used primary and established cell line. Established cell lines have the advantage of enhanced reproducibility of results and are recommended by the ISO for preliminary cytotoxicity screening. For specific sensitivity testing in order to stimulate the in vivo situation, primary cell strain derived from live tissue are necessary and also recommended by ISO (9).

Adhesion and spreading of cells on a material surface are the initial phase of cellular function.

The major events in this phase are the attachment of cell to the substratum, radial growth of filopodia, cytoplasmic webbing, and resultant flatting of cell (9). In general positive and MTA and Portland cement groups, attachment on surface and normal morphology were observed. The persistent of rounded cells with little or no spreading suggested that the surface material might be toxic. On surface of negative group (IRM) cells were totally rounded without attachment. Such toxic products affect both the morphology and attachment behavior of the cells.

The results of our study was in general agreement with Perez and Spears in 2003 that observed normal morphology and adhesion of MG-63 cell line after 9 days (5) and with Koh (8) in 1997 and Zhu (1) that showed in presence of MTA osteoblast were attached with normal morphology.

Based on the results of our study, response of cells in presence of white MTA and Portland cement was similar and showed that both materials are biocompatible. Saidan *et al.* and Abdullah *et al*. evaluated the biocompatibility of two variant of accelerated Portland cement (APC) in-vitro by observing the cytomorphology of saos-2 osteosarcoma cells. Glass ionomer cement, MTA and unmodified Portland cement (PC) were used for comparison. On SEM evaluation, healthy Saos2 cells were found adhering to the surfaces of APC variant, PC and MTA that was in agreement with our results (10,11). De Deus *et al.* evaluated the cytotoxic effects of gray and white MTA and Portland cement used the human ECV 304 endothelial cell line. Effect of the materials on mitochondrial functions was measured by a colorimetric assay. No statistically significant difference was shown between any of the experimental materials. The two brands of MTA as well as Pc initially showed a similar elevated cytotoxic effect that decreased gradually with time allowing the cell culture to become reestablished (12).

Genotoxic and cytotoxic effects of MTA and Portland cement were evaluated in-vitro using alkaline single cell gel (comet) assay and trypan blue exclusion test on mouse lymphoma cells by Ribeiro and *et al.* in 2005. The results of their study demonstrated that the single cell gel assay failed to detect DNA damage after treatment of cells by MTA and Portland cement. Similar result showed that none of the compound tested were cytotoxic and genotoxic (13).

The difference in adhesion of osteoblasts to different materials can be related to different surface roughness or in the chemical composition of materials (5).

## CONCLUSION

Under condition of the results of this study responses of osteoblasts in the presence of white MTA and Portland cement were similar and both materials were biocompatible and using of these materials in adjacent vital tissue is not toxic and these materials can promote healing in priapical tissue.
